# Acute Brainstem Dysfunction Caused by Cavernous Sinus Dural Arteriovenous Fistula

**DOI:** 10.1155/2020/2630959

**Published:** 2020-05-23

**Authors:** Yuwa Oka, Kenichi Komatsu, Soichiro Abe, Naoya Yoshimoto, Junya Taki, Sadayuki Matsumoto

**Affiliations:** ^1^Department of Neurology, Kitano Hospital, Tazuke Kofukai Medical Research Institute, Osaka, Japan; ^2^Department of Neurosurgery, Kitano Hospital, Tazuke Kofukai Medical Research Institute, Osaka, Japan

## Abstract

Symptoms of cavernous sinus dural arteriovenous fistula depend on the drainage patterns and are very diverse. Among these, brainstem dysfunction is a rare but serious complication. Here, we describe a case with isolated and rapidly progressive brainstem dysfunction due to cavernous sinus dural arteriovenous fistula. An 80-year-old woman presented with a 2-day history of progressive gait disturbance. Neurological examination revealed mild confusion, dysarthria, and left hemiparesis. Magnetic resonance imaging (MRI) revealed pontine swelling without evidence of infarction. Magnetic resonance angiography suggested a faint abnormality near the cavernous sinus. Dural arteriovenous fistula was suspected, and digital subtraction angiography was planned for the next day. Her condition had progressed to coma by the next morning. Pontine swelling worsened, and hyperintensity appeared on diffusion-weighted imaging. Digital subtraction angiography revealed a right-sided cavernous sinus dural arteriovenous fistula with venous reflux into the posterior fossa. Orbital or ocular symptoms had preceded brainstem symptoms in all nine previously reported cases, but brainstem symptoms were the only presentation in our case, making the diagnosis difficult. Some dural arteriovenous fistulas mimic inflammatory diseases when the clinical course is acute. Prompt diagnosis using enhanced computed tomography or MRI and emergent treatment are needed to avoid permanent sequelae.

## 1. Introduction

Symptoms of cavernous sinus dural arteriovenous fistula depend on the drainage patterns and are very diverse. Among these, brainstem dysfunction is a rare but serious complication. Here, we describe a case with isolated and rapidly progressive brainstem dysfunction due to cavernous sinus dural arteriovenous fistula.

## 2. Case Presentation

**Figure 1:**
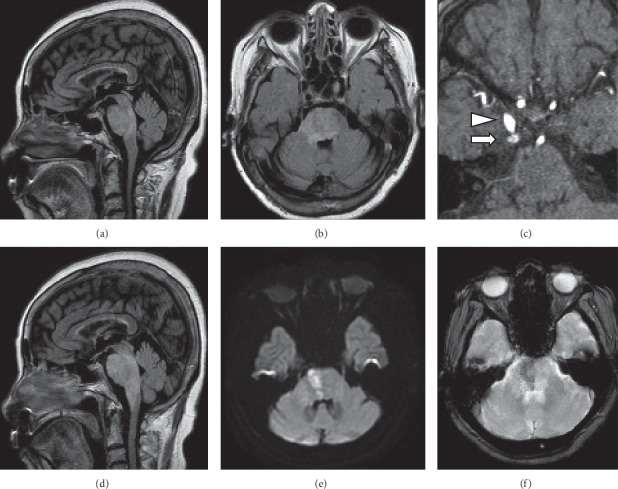
Magnetic resonance imaging. (a, b) Fluid-attenuated inversion recovery (FLAIR) imaging showing widespread hyperintensity diffusely involving the right pons on day 1. (c) Magnetic resonance angiography showing an abnormal flow signal (arrow) posterolateral to the cavernous sinus. Arrowhead indicates the right internal carotid artery. (d) FLAIR imaging. Brainstem edema has worsened and expanded rostrocaudally on day 2. (e) Diffusion-weighted showing shows hyperintense lesion within the edematous pons. (f) T2 star-weighted imaging showing an area of low intensity, suggesting venous congestion and hemorrhage.

**Figure 2:**
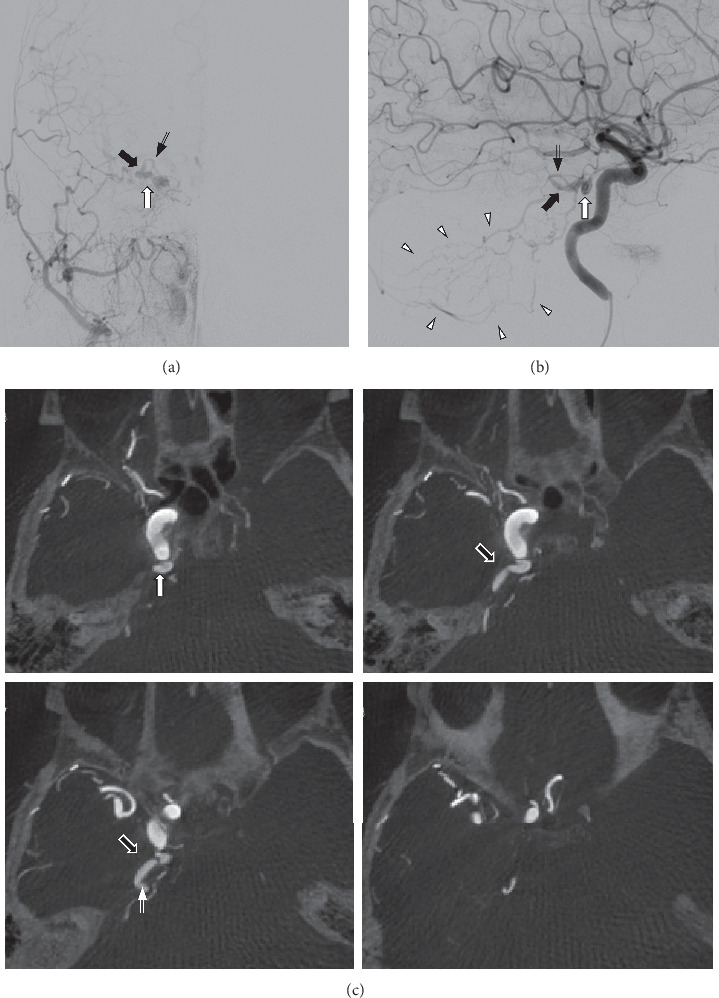
(a) Anteroposterior view of the right external carotid artery angiogram and (b) lateral view of the right internal carotid artery angiogram demonstrate a right-sided cavernous sinus dural arteriovenous fistula (white arrow) draining into the superior petrosal sinus (black arrow) and petrosal vein (double arrow). Note the retrograde venous drainage into the brainstem and cerebellar cortical veins (arrowheads). (c) Axial maximum intensity projection images of the right internal carotid artery angiogram. White arrow indicates the cavernous sinus dural arteriovenous fistula. No bridging veins other than the superior petrosal sinus (black arrow) and petrosal vein (double arrow) are detected.

We report the case of an 80-year-old woman who presented to the neurology department with a 2-day history of progressive gait disturbance. Neurological examination revealed mild confusion, dysarthria, and left hemiparesis. She needed assistance to stand and could not walk unassisted. Magnetic resonance imaging (MRI) revealed fluid-attenuated inversion recovery (FLAIR) hyperintensity, dominant on the right side (Figures 1(a) and 1(b)). Diffusion-weighted imaging (DWI) showed no significant hyperintensity. Magnetic resonance angiography suggested a faint flow signal abnormality posterolateral to the cavernous sinus (CS) (Figure 1(c)). Dural arteriovenous fistula (dAVF) was suspected, and we planned digital subtraction angiography (DSA) for the next day. High-dose methylprednisolone was administered, considering the possibility of inflammatory disease. The next morning, her condition had progressed to coma. Pontine swelling had worsened (Figure 1(d)), and DWI hyperintensity had appeared (Figure 1(e)). T2 star-weighted imaging showed an area of low intensity (Figure 1(f)), suggesting venous congestion and hemorrhage. DSA revealed a right-sided CS dAVF with venous reflux into the brainstem and cerebellar cortical veins *via* the right superior petrosal sinus and petrosal vein (Figures 2(a)–2(c)). We attempted transvenous and transarterial embolizations, but could not approach close to the shunt pouch. Another approach was to surgically close the petrosal vein with a craniotomy. However, respiratory failure developed, and the patient was intubated during the transcatheter procedure. As a poor prognosis was predicted by severe neurological deficit and high age, we refrained from further intervention. Strict antihypertensive and antiedema therapies were initiated. MRI after 5 days showed spontaneous occlusion of the fistula. T2 star hypointensity at the pons persisted, and the hemorrhagic infarction was confirmed. Her level of consciousness gradually improved, and she was transferred for rehabilitation 3 months later, with severe weakness of the left extremities.

## 3. Discussion

Symptoms of CS dAVF depend on the drainage patterns and are very diverse, including orbital, ocular, cranial nerve, and cerebral symptoms. Cerebral symptoms occur in 3–5% of cases [1, 2]. Among these, brainstem dysfunction is a rare but serious complication. In our case, major drainage routes had been obliterated, and the right CS was isolated. Previous reports have pointed that increased venous pressure causes the thickening of the intima of the affected sinus and other draining vessels and can obliterate the drainage routes [3, 4]. We could not find direct evidence of a thrombus in the cavernous sinus or superior ophthalmic vein, but probably obliteration occurred as a natural history of dAVFs. Compartmentalizaion of the CS may have played a role. Single drainage route *via* the right petrosal vein caused venous reflux into posterior fossa, and subsequent focal venous congestion caused acute brainstem dysfunction and hemorrhagic infarction. Orbital or ocular symptoms had preceded brainstem symptoms in all nine cases previously reported in the English literature [5–10], but brainstem symptoms were the only presentation in our case, making the diagnosis difficult.

Some dAVFs lack orbital or ocular symptoms and mimic inflammatory diseases when the clinical course is acute. Prompt diagnosis using enhanced CT or MRI and emergent treatment are needed to avoid permanent sequelae.

## References

[1] Suh D. C., Lee J. H., Kim S. J. (2005). New concept in cavernous sinus dural arteriovenous fistula. *Stroke*.

[2] Kiyosue H., Hori Y., Okahara M. (2004). Treatment of intracranial dural arteriovenous fistulas: current strategies based on location and hemodynamics, and alternative techniques of transcatheter embolization. *Radiographics*.

[3] Misaki K., Uchiyama N., Mohri M. (2009). Unique venous drainage of a sphenoid wing dural arteriovenous fistula with ocular symptoms. *Neuroradiology*.

[4] Nishijima M., Takaku A., Endo S. (1992). Etiological evaluation of dural arteriovenous malformations of the lateral and sigmoid sinuses based on histopathological examinations. *Journal of Neurosurgery*.

[5] Miyagishima T., Inoue M., Ohno H. (2012). Pontine venous congestion due to dural arteriovenous fistula of the cavernous sinus: case report and review of the literature. *Surgical Neurology International*.

[6] Uchino A., Kato A., Kuroda Y., Shimokawa S., Kudo S. (1997). Pontine venous congestion caused by dural carotid-cavernous fistula: report of two cases. *European Radiology*.

[7] Takahashi S., Tomura N., Watarai J., Mizoi K, Manabe H (1999). Dural arteriovenous fistula of the cavernous sinus with venous congestion of the brain stem: report of two cases. *AJNR. American Journal of Neuroradiology*.

[8] Shintani S., Tsuruoka S., Shiigai T. (2000). Carotid-cavernous fistula with brainstem congestion mimicking tumor on MRI. *Neurology*.

[9] Kai Y., Hamada J.-I., Morioka M., Yano S., Ushio Y. (2004). Brain stem venous congestion due to dural arteriovenous fistulas of the cavernous sinus. *Acta Neurochirurgica*.

[10] Iwasaki M., Murakami K., Tomita T., Numagami Y., Nishijima M. (2006). Cavernous sinus dural arteriovenous fistula complicated by pontine venous congestion. A case report. *Surgical Neurology*.

